# Integrating Mobile-health, health coaching, and physical activity to reduce the burden of chronic low back pain trial (IMPACT): a pilot randomised controlled trial

**DOI:** 10.1186/s12891-019-2454-y

**Published:** 2019-02-11

**Authors:** Anita B. Amorim, Evangelos Pappas, Milena Simic, Manuela L. Ferreira, Matthew Jennings, Anne Tiedemann, Ana Paula Carvalho-e-Silva, Eduardo Caputo, Alice Kongsted, Paulo H. Ferreira

**Affiliations:** 1 Discipline of Physiotherapy, Faculty of Health Sciences, The University of Sydney, Sydney, Australia; 2 Institute of Bone and Joint Research, The Kolling Institute, Sydney Medical School, Sydney, Australia; 3 Physiotherapy Department, Liverpool Hospital, South Western Sydney Local Health District, Sydney, Australia; 4 School of Public Health, The University of Sydney, Sydney, Australia; 5 Postgraduate Program in Physical Education, Federal University of Pelotas, Pelotas, Brazil; 6 Department of Sports Science and Clinical Biomechanics, University of Southern Denmark, Denmark. Nordic Institute of Chiropractic and Clinical Biomechanics, Odense, Denmark; 7 Faculty of Health Sciences, The University of Sydney, 75 East Street, Lidcombe, Sydney, NSW 1825 Australia

**Keywords:** Physical activity, Low back pain, Mobile health, Health coaching, Randomized controlled trial

## Abstract

**Background:**

Low back pain is one of the most prevalent musculoskeletal conditions and the highest contributor to disability in the world. It is characterized by frequent relapses leading to additional care-seeking. Engagement in leisure physical activity is associated with lower recurrences and better prognosis and potentially reduced care-seeking. Our aim was to investigate the feasibility and preliminary efficacy of a patient-centred physical activity intervention, supported by health coaching and mobile health, to reduce care-seeking, pain and disability in patients with chronic low back pain after treatment discharge.

**Methods:**

We conducted a pilot randomised controlled trial with blinded outcome assessment. Sixty-eight participants were recruited from four public outpatient physiotherapy departments and the general community in Sydney. The intervention group received a physical activity information booklet, plus one face-to-face and 12 telephone-based health coaching sessions. The intervention was supported by an internet-based application and an activity tracker (*Fitbit*). Control group (standard care) received the physical activity information booklet and advice to stay active. Feasibility measures included recruitment rate, intervention compliance, data completeness, and participant satisfaction. Primary outcomes were care-seeking, pain levels and activity limitation. Outcomes were assessed at baseline, 6-month follow-up and weekly for 6 months.

**Results:**

Ninety potential participants were invited over 15 months, with 68 agreeing to take part (75%). Overall, 903 weekly questionnaires were answered by participants from a total of 1107 sent (89%). Participants were largely satisfied with the intervention (mean = 8.7 out of 10 on satisfaction scale). Intervention group participants had a 38% reduced rate of care-seeking (Incidence Rate Ratio (IRR): 0.62, 95% CI: 0.32 to 1.18, *p* = 0.14, using multilevel mixed-effects Poisson regression analysis) compared to standard care, although none of the estimates was statistically significant. No between groups differences were found for pain levels or activity limitation.

**Conclusion:**

The health coaching physical activity approach trialed here is feasible and well accepted by participants and may reduce care-seeking in patients with low back pain after treatment discharge, although further evaluation with an adequately powered trial is needed.

**Trial registration:**

Australian and New Zealand Trial Registry ACTRN12615000189527. Registered prospectively on 26–02–2015.

**Electronic supplementary material:**

The online version of this article (10.1186/s12891-019-2454-y) contains supplementary material, which is available to authorized users.

## Background

Low back pain (LBP) is the leading cause of disability worldwide. [1] It is a common condition that affects more than 500 million people globally at any one time. [2] LBP is typically recurrent, with 24 to 87% of individuals who experience an episode of LBP suffering a recurrent episode within one year. [3–5] In many instances, people with activity-limiting LBP experience recurrent episodes that may be longer in duration and be associated with higher levels of disability. This often results in high healthcare utilisation and prolonged time-loss from work, incurring AUD $9 billion in direct and indirect costs to Australia’s economy. [6–8]

While there is evidence that conservative interventions, such as exercise and spinal manipulative therapy, improve short-term pain and disability in people with chronic LBP, [9, 10] patients typically exhibit a rapid decline in clinical outcomes after treatment discharge, [9, 11] and further care-seeking for LBP is common. [12] For instance, a recent longitudinal study conducted in Denmark involving 1082 participants presenting with non-specific LBP to general practitioners (GP) and chiropractors showed that over a 1-year follow-up period people still report having mild to moderate LBP (mean intensity of 3 on a 0–10 pain scale), on an average of 3 days per week. [12] Likewise, 32% will seek additional care (e.g. GP visit) over the course of 5 years after primary care treatment [13].

A potential contributor to clinical decline is lack of adherence and motivation to maintain physical activity levels as recommended by LBP self-management guidelines. [14] It has been suggested that leisure-time physical activity has a positive impact on the course of LBP. [15] For instance, people with chronic LBP who are physically active experience less pain (− 0.6, 95% CI: − 1.0 to − 0.1; 0–10 numerical pain scale) and disability (− 8.7, 95% CI: − 14.2 to − 3.1; 0–100 disability scale) than those not maintaining adequate levels of physical activity. [16] However, most people with chronic LBP tend to become more sedentary during their leisure time than those without chronic LBP. [17, 18] According to qualitative studies exploring the experiences, opinions, and treatment expectations of chronic LBP patients, [19, 20] although patients recognise the value of advice and exercise, they usually mistrust the appropriateness of this approach given the fact that a precise diagnosis of their condition is rarely given, and symptoms often recur. [19] Consequently, poor adherence to advice and physical activity seems to be the primary factor limiting the potential effectiveness of long-term active self-management strategies for chronic LBP. [19] A systematic review of 20 high-quality cohort studies found substantial evidence suggesting that poor treatment adherence was correlated to low levels of physical activity at baseline, low self-efficacy, depression, anxiety, insufficient social support/activity, more perceived barriers to exercise and increased pain levels during exercise. [14] Therefore, interventions aimed at supporting people with LBP to engage in active lifestyles after treatment discharge should be encouraged.

Patient-centred approaches, supported by shared decision making, are usually more effective than general, non-specific approaches for promoting behaviour change, such as engagement in physical activity for people with non-specific chronic LBP. [21–24] A recent systematic review demonstrated that patient-centred approaches, such as motivational interventions, are effective at increasing physical activity behaviour for different clinical populations, including people with LBP. [25] Health coaching is based on behaviour change theory and aims to encourage and support healthier lifestyle choices. [26–28] There is strong evidence that health coaching can positively impact on health behaviours, including physical activity, [29] nutrition, [30] smoking cessation, [31] and self-management of chronic conditions. [32] Additionally, Mobile health (m-Health) technologies, such as internet-based platforms (e.g. web applications, websites) are increasingly used to support behaviour change. M-Health has the potential to increase accessibility of treatment through the delivery of convenient, individually tailored, and contextually meaningful behavioural interventions. [33–35] Likewise, physical activity trackers (e.g. *Fitbits*) are effective in promoting physical activity uptake in people with musculoskeletal conditions, including LBP. [36, 37] However, the effectiveness of health coaching in addition to m-Health technologies to increase physical activity levels and improve health outcomes in a population with chronic LBP after treatment discharge is unknown.

Therefore, we designed a pilot trial to test the feasibility and preliminary efficacy of a patient-centred physical activity intervention, supported by health coaching and m-Health technology to reduce care-seeking, pain and activity limitation in patients with chronic LBP after discharge from conservative treatment, compared to standard care. The secondary aim was to examine the effect of this intervention on physical activity adherence and goal attainment.

## Methods

### Study design

The trial protocol has been published in detail elsewhere [38] and is summarised briefly here. We conducted a pilot randomised controlled trial with blinded outcome assessment.

The trial is reported in accordance with the CONSORT guidelines for clinical trials [39] and the intervention is reported in accordance with the TIDieR checklist for reporting of interventions. [40] The Human Research Ethics Committee from the South Western Sydney Local Health District approved this study (project number: 15/015). Participants gave written informed consent before data collection began.

### Participants

Participant recruitment was conducted between March 2016 and July 2017. Participants were recruited from outpatient physiotherapy departments of four public hospitals and from the general community in Sydney metropolitan area. This was a deviation from the protocol where we proposed recruitment from a single hospital. Due to low recruitment rate we expanded our recruitment to patients who met the inclusion criteria in three additional hospitals as well as the general community. To expand the recruitment to the general community we amended the inclusion criteria to include people that were discharged not only from a hospital-based physiotherapy treatment but also from private practices (e.g. physiotherapy, chiropractic or GP). At the hospitals, individuals were invited to participate in the trial by their treating physiotherapists. Participants from the general community were invited through newsletters (e.g. Seniors Cards’ newsletter), or social media (e.g. Facebook).

Individuals who expressed interest in participating in the study were contacted by the research team and screened for eligibility, according to the following eligibility criteria: i) aged 18 years or older; ii) reported chronic LBP persisting for over 12 weeks; iii) discharged from physiotherapy but still symptomatic (score at least 3 on a 0–10 Numerical Pain Scale); iv) regular (weekly) users of a computer or internet-connected mobile/tablet device; and v) fluent in English (verbal and written). Exclusion criteria included: i) pregnancy; ii) diagnosis of serious spinal pathology (e.g. metastatic, inflammatory, or infectious diseases of the spine); iii) a history of spinal surgery in the past 12 months; iv) fibromyalgia, or systemic/inflammatory disorder; v) comorbid health conditions that would prevent active participation in increasing physical activity levels: cardio-respiratory illnesses; or vi) LBP caused by involvement in a road traffic accident in the last 12 months or ongoing litigation.

Patients who met the inclusion criteria at the hospital were given a pamphlet about the study. Potential participants interested in participating in the study were referred to the research team. Patients could either contact the research team directly through the phone number provided in the pamphlet or be contacted by the research team via their contact details provided by the hospital staff. Patients who agreed to participate arranged a date and time to see a study investigator at the physiotherapy department soon after discharge. On the assigned date and time, a signed consent form was recorded, and baseline data were collected. People from the general community went through a slightly different process, if they were interested in the trial they could contact the research team via e-mail or via phone. Screening of potential participants from the general community was accomplished by e-mail or over the phone by a study investigator. If they met the inclusion criteria and had been recently discharged from treatment for their back pain (e.g. exercise therapy, spinal manipulative therapy), they were invited to see a study investigator at the University of Sydney where the research team was based.

### Assessments

Once consent was obtained, a study investigator collected anthropometric (e.g. age, height, weight), and demographic data (e.g. education level, smoking status), as well as trial baseline data, through self-reported questionnaires embedded in an electronic platform (hosted at the University of Sydney), created specifically for the study. The baseline assessment was completed face-to-face on the same day that the participant was recruited, with the questionnaire being completed on the research team’s iPad. Participants were able to ask any question regarding the questionnaire to a study investigator in case they had an enquiry. Objective assessment of physical activity was performed with a triaxial accelerometer (*Actigraph GT3X+*). *Actigraph* has been widely used in clinical research, and it has been shown to be valid for discriminating levels of physical activity in different populations, including people with LBP. [41–46]

The weekly follow-up surveys were sent to participants via an electronic link embedded in a mobile text message or e-mail. No reminders were sent after the weekly survey; however, if the participant did not respond to the survey for four consecutive weeks, the study administrator received a reminder to contact the participant. One week before the end of the 6-month intervention, a study investigator contacted the participant to arrange a time to conduct the 6-month follow-up assessment, which was also completed online on the investigator’s iPad. If the participant was not able to meet to study investigator, the questionnaire was sent via e-mail, and the *Actigraph* was sent to the participant’s address via post.

### Randomisation and blinding

Randomisation was performed in a 1:1 ratio to the active intervention or standard care group. To ensure allocation concealment, randomisation to groups was undertaken by a blinded remote investigator (MS) not involved in recruitment using a computer-generated random number schedule of 10 permuted blocks of 6 and the final block of 8. Study investigators conducting data collection were blinded to group allocation.

### Intervention group

The intervention group received a physical activity and sedentary behaviour information booklet developed by the Australian Government Department of Health called ‘Make your move – Sit less, be active for life’ [47] and advice to stay active delivered by the study investigator right after baseline completion and before randomisation. In addition, after randomisation, participants developed an individually tailored physical activity plan with guidance from a health coach, who was trained through the Wellness Coaching Australia course. Each participant received an initial home-based face-to-face coaching session that lasted between 1 and 2 h, that included motivational interviewing and solution-focused goal setting. [26] The focus of the patient-centred physical activity plan was to motivate and support participants to gradually increase their leisure-time and incidental physical activity. Participants were encouraged to devise fortnightly goals to suit and advance their physical activity levels. After the first face-to-face coaching session, the health coach contacted participants fortnightly (12 phone calls per participant over 6 months) to assess progress, update short-term goals, and assist in overcoming barriers.

**Fig. 1:**
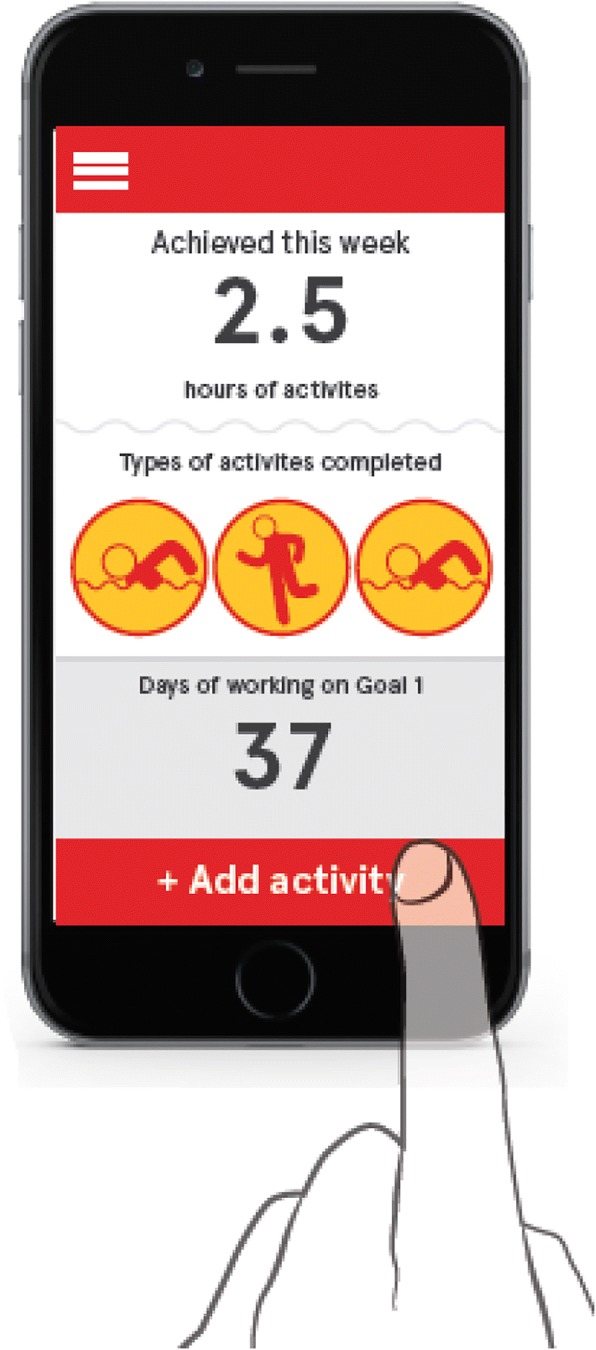
IMPACT web app

This intervention was also supported by an activity tracker (*Fitbit*), and a specifically designed mobile web application (IMPACT app) (Fig. 1). Participants were able to access the IMPACT app at any time to monitor their goals and physical activities and report on physical activity-related goals. The health coach used the participant reports to guide the telephone coaching sessions, discuss participants’ goals, and progress. Personalised messages, referred to as “healthy tips”, were sent on a weekly basis to encourage participants to achieve their goals. The intervention details are included in Table 1.

**Table 1:** Intervention description using the Template for Intervention Description and Replication (TIDieR) checklist

1. Brief name	Integrating Mobile-health, health coaching, and Physical Activity to reduce the burden of Chronic low back pain Trial (IMPACT)
2. Why	Low back pain is a significant public health problem and engagement in moderate levels of physical activity is associated with positive outcomes. Conservative active care, such as exercise, is effective in reducing pain and disability associated with chronic low back pain. However, a rapid decline in clinical outcomes is commonly seen after discharge from treatment. These problems need to be urgently addressed as the burden of low back pain continues to rise.
3. What- materials	• The “Make your move – Sit less, be active for life” physical activity booklet developed by the NSW Ministry of Health • A specifically designed mobile web application (IMPACT app) to monitor participants’ goals and physical activities. • A pedometer enhanced with a web-interface (“fitbit”, www.fitbit.com/au) to give feedback on the amount of daily physical activity achieved.
4. What- procedures	Telephone or email-based health coaching was used to identify barriers and facilitators to physical activity participation, and to provide education and support to assist participants to achieve their physical activity goals.
5. Who provided	Three health coaches with professional backgrounds in physiotherapy and exercise physiology delivered the intervention.
6. How	The health coaching was delivered during one face to face session plus 12 fortnight telephone-based sessions.
7. Where	The intervention was delivered to people with chronic low back pain after discharge from treatment from hospitals and general community in Sydney and surrounds, Australia.
8. When and how much	The face to face assessment and interview occurred at the beginning of the intervention period and lasted for approximately 2 h. The telephone-based health coaching occurred after the face to face assessment and interview, once every 2 weeks for approximately 20 min for a total duration of 6 months.
9. Tailoring	The physical activity plan was tailored to participant goals, current physical ability and preferences.

### Control group

The control group received the ‘Make your move – Sit less, be active for life!’ [47] booklet and brief advice to stay active which was delivered right after baseline completion and before randomisation by a study investigator. After randomisation, participants received an advice to work independently towards increasing their physical activity levels and achieving their two long-term goals as defined at baseline, which was delivered once, over the phone, by a study investigator.

### Sample size calculation

We estimated that a sample size of 68 participants would provide 80% power to detect a 2-point between-group difference on the pain levels outcome measured by the Pain Numerical Rating Scale, assuming a standard deviation of 1.9 points. We anticipated a maximum dropout rate of 35% and alpha of 5%. [48]

### Assessment of feasibility

#### Recruitment

Records were kept of the number of individuals screened for eligibility, the number eligible and invited to participate and the number that consented to participate per recruitment site. When available, the reasons for not entering the study were also recorded. Our recruitment metric was calculated based on the number of participants consenting to participate in the study as a proportion of participants invited.

#### Measures of completeness of data collection and compliance

The number of participants who responded and provided valid data for each weekly follow-up of primary outcomes (completeness of data) was recorded. This is a crucial aspect of data quality and an important measure as we collected data electronically on a weekly basis. We assessed completeness of data collected for each of the outcome measures, with > 80% valid data used as criteria to consider the study feasible. We also recorded who complied with the accelerometer (*Actigraph*) protocol (compliance). Compliance with the accelerometer protocol was considered valid if the participant wore the device for at least 10 h a day for at least four days. [49] A previously established algorithm (Choi 2011) was used to determine ‘non-wear’ time. [50] Participant’s compliance with the intervention was also measured by the number of health coaching sessions completed.

#### Barriers and facilitators to completing the study

A semi-structured interview with intervention participants who completed the study was conducted to gather data on their experience, and the barriers and facilitators to participation. Participants also rated their experiences regarding the health coaching, use of the *Fitbit,* and the IMPACT web app, using open-ended questions as well as Likert-based scales. In this study, only quantitative data from the interviews are presented. Qualitative data will be presented in a future publication.

### Assessment of intervention impact

#### Primary outcomes

Primary clinical outcomes were care-seeking, pain levels and activity limitation, collected weekly during the 6 months of the intervention through a study-specific electronic survey that included questions about LBP (Additional file 1). An episode of care-seeking was defined as a consultation or a series of consultations for LBP based on the definition that has been suggested by de Vet et al. [51] Consultations for LBP included visiting a GP, a physiotherapist, a chiropractor, emergency department or surgical procedure. Self-management (e.g. medication, heat pack) was also considered as care-seeking. Pain levels were assessed with an 11-point scale ranging from 0 to 10, where 0 defines the absence of pain and 10 describes unbearable pain, the numerical rating scale (NRS). [52] Although in the protocol of this study, we refer to one of the primary outcomes as disability, we have renamed this outcome to activity limitation which is more suitable to our research question. This was a protocol deviation. Activity limitation was based on the question: “Was the low back pain bad enough to limit your usual activities in the last 7 days?”, and it was assessed weekly over 6 months. Disability was also assessed using the Roland–Morris Disability Questionnaire (RMDQ; range 0–24) [53] but only at baseline and 6-month follow-up and is presented as a separate outcome.

#### Secondary outcomes

Secondary outcomes were assessed at baseline and 6-month follow-up. Self-reported physical activity was assessed using the International Physical Activity Questionnaire (IPAQ; minutes of engagement in physical activity per week). [54] Physical activity was assessed objectively over a 7-day period using the accelerometer (*Actigraph* GT3X+). To be considered a valid day, wear time was defined as 10 h or more on four or more days, and non-wear time was defined as 90 min of consecutive zero counts. This was a deviation of the protocol determined to improve the quality of the *Actigraph* data as recently published studies [55–57] have shown that a 90-min non-wear time window is more sensitive and specific when compared to a 60-min non-wear time window when analysing 24 h-accelerometry. *Actigraphs* were initialised to collect triaxial acceleration data using a frequency of 30 Hz, and data were aggregated to 60-s epochs using Actilife software 6.13.3. Physical activity data were summarised to produce measures of overall light physical activity, moderate-to-vigorous physical activity (MVPA) and average step count per week. [58] This was a deviation from the protocol in order to aid interpretation based on the World Health Organization (WHO) physical activity guideline recommendation of 150 min of MVPA per week. [59] Goal attainment was assessed using the Goal Attainment Scale (GAS; range − 2 to 2). [60] Participants were requested, at baseline, to set two goals (related to physical activity) to be achieved within 6 months and the degree of goal attainment was assessed at 6 months.

### Other variables

Other variables, such as fear avoidance beliefs, emotional states of depression, anxiety and stress, and sleep quality have been collected and is reported at baseline only. Fear avoidance was assessed using the Fear-Avoidance Beliefs Questionnaire (FABQ; range 0–96). There are two subscales within the FABQ; the work subscale (FABQw) with 7 questions (range 0–42) and the physical activity subscale (FABQpa) with 4 questions (range 0–24). [61] Emotional states of depression, anxiety and stress was assessed using the Depression Anxiety Stress Scale (DASS; range 0–21 each domain). [62] Sleep quality was evaluated using item 6 from the Pittsburgh Sleep Quality Index (PSQI) [63] which evaluates sleep quality based in four categories (very bad, fairly bad, fairly good, very good).

### Data analysis

Descriptive statistics were used to describe the baseline characteristics of included participants. We used multilevel mixed-effects models to calculate between-group incidence rate ratios (IRR) for the number of episodes of care-seeking, and activity limitation per person throughout the follow-up period (6 months) using Poisson regression. We estimated the group effect over time by fitting an interaction term between group and time. We also used the multilevel mixed-effects model, taking into account individual follow-up time, the frequency of events, non-normal distribution of data over time, and non-independence of repeated measures. Pain intensity was analysed as a continuous repeated variable using a multilevel mixed linear regression model with random intercepts. The effect of group allocation at single time points (baseline and 6-month follow-up) on continuous outcomes (e.g. disability and physical activity) was assessed using linear regression models. We analysed between-group differences in mobility-related goal attainment at 6 months. To aid interpretation of the GAS, the scores were dichotomised (goal met versus goal not met), and odds ratios calculated. For the analysis, we have chosen the highest GAS score. For instance, if the goal one was scored as “0” and the goal two as “-1”, we have used “0” for the analysis. This was a protocol deviation. All analyses were performed by intention to treat. Stata IC 12.0 (StataCorp Texas, USA) was used for analyses.

## Results

### Flow of participants through the study

**Fig. 2:**
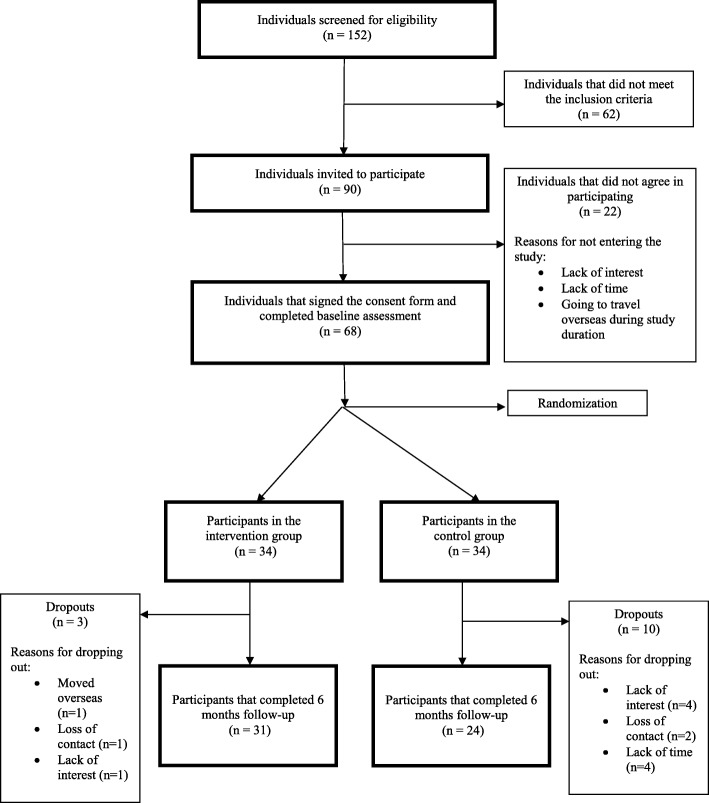
Design and flow of participants through the study

The flow of participants through the study is shown in Fig. 2. In total, 152 potential participants were screened for eligibility, from those 90 met the inclusion criteria (59%) and were invited to participate, with 68 agreeing to participate (75%). Of these, 33 participants were recruited over 12 months following discharge from physiotherapy treatment in outpatient departments of four public hospitals; and 35 participants were recruited over three months from the general community (51%). Thirty-four participants were randomised to one of the two groups. Most people from the hospital sites that did not meet the inclusion criteria were excluded because they did not speak English, as opposed to people from the general community that were excluded because they had not previously had physiotherapy treatment for their chronic LBP. Follow-up data were collected from 31 intervention group participants (3 dropouts), and from 24 control group participants (10 dropouts), with a total of 19% of loss to follow-up.

### Characteristics of participants

Participant characteristics are described in Table 2. The two groups did not differ significantly on demographic factors, (mean age was 58.4 SD ± 13.4, and 50% were female), however, there was a 12% difference in gender between the groups, with the intervention group containing fewer females than the control group. Most participants were non-smokers (58%) and were considered overweight [Body Mass Index (BMI) mean 28.0 SD ± 5.5]. Participants assigned to the intervention and control groups were similar regarding pain levels and disability. Participants in the intervention group reported slightly higher baseline self-reported MVPA (minutes per week) when compared to the control group (mean = 199.1, SD ± 672.2; mean = 129.8, SD ± 392.2, respectively). However, objective measures of physical activity revealed similar results between groups (e.g. average time spent in MVPA was 197.5, SD ± 141.1; and 209.0, SD ± 170.5, for the intervention and control groups respectively). Nevertheless, the total minutes of physical activity per week measured with the *Actigraph* was higher in the intervention group when compared with the control group (mean = 2241, SD = 886; mean = 2099, SD = 842, respectively).

**Table 2:** Baseline characteristics of the IMPACT study population divided by group, numbers are mean (SD) unless otherwise stated

Variables	Intervention group (*n* = 34)	Control group (*n* = 34)
Age	59.5 (11.9)	57.1 (14.9)
Gender (female), number (%)	15 (44)	19 (56)
Body mass index	28.9 (6.0)	27.2 (5.1)
Non-smoker, number (%)	18 (53)	21 (62)
Education (graduate), number (%)	11 (32)	12 (35)
Pain intensity^a^	5.4 (1.7)	5.2 (1.7)
Disability^b^	8.9 (5.4)	9.0 (6.1)
Self-reported total Physical activity^c^	609 (886)	625 (812)
Self-reported MVPA^d^	199.1 (672.2)	129.8 (392.2)
Objective total Physical activity^e^	2241 (886)	2099 (842)
Objective MVPA^f^	197.5 (141.1)	209.0 (170.5)
Moderate-to-vigorous physical activity^g^, number (%)	18 (53)	11 (32)
Fear avoidance beliefs^h^, number (%)	21 (62)	22 (65)
Depression^i^	3.5 (4.9)	2.8 (3.7)
Anxiety^i^	2.9 (3.1)	2.1 (2.1)
Stress^i^	4.9 (3.4)	3.7 (3.5)
Fairly good sleep quality^j^, number (%)	16 (47)	14 (41)

^a^Pain intensity measured with the numerical rating scale (0–10)

^b^Disability measured with the Rolland-Morris Disability Questionnaire (0–24)

^c^Total minutes of physical activity per week measured with the International Physical Activity Questionnaire (IPAQ)

^d^Engagement moderate-to-vigorous physical activity (MVPA) measured with the IPAQ

^e^Total minutes of physical activity per week measured with the *Actigraph*

^f^Engagement in MVPA measured with the *Actigraph*

^g^Percentage of participants meeting the physical activity guidelines (at least 150 min of moderate to vigorous physical activity)

^h^Percentage of participants presenting high score in the fear avoidance belief questionnaire (FABQ) subscale for physical activity

^i^Mean scores for Depression, Anxiety and Stress collected with the Depression, Anxiety and Stress Scale (DASS)

^j^Percentage of patients self-reporting fairly good sleep quality based on item 6 from the Pittsburg Sleep Quality Index (PSQI)

### Completeness of data collection and compliance

Unfortunately, there were some technical issues with the web-based application used to collect outcomes which influenced the number of weekly surveys sent to the participants. For this reason, not every participant received the same number of surveys, however, on average, participants received 19 weekly surveys in total over the 6 months. From the surveys sent, on average 16 were completed. Overall, 903 weekly questionnaires were answered by participants from a total of 1107 sent (89%), which was above the cut-off point established to test the feasibility of the study (80%). From those, 469 (52%) weekly surveys were completed by participants in the intervention group (mean = 15 weekly surveys completed per participant, IQR = 15), and 434 (48%) were completed by participants in the control group (mean = 16, IQR = 22). Overall, there were 20% of missing data at the 6-month questionnaire follow-up and 16% of missing data across the 6-month weekly surveys. *Actigraph* data were collected for 48 of the 68 participants (71%). From those, 28 (58%) participants were from the intervention group, and 20 (42%) participants were from the control group. The remaining participants failed to wear the *Actigraph* for at least four days as required and the data collected were insufficient for analysis.

### Participants’ experiences with the intervention

**Fig. 3:**
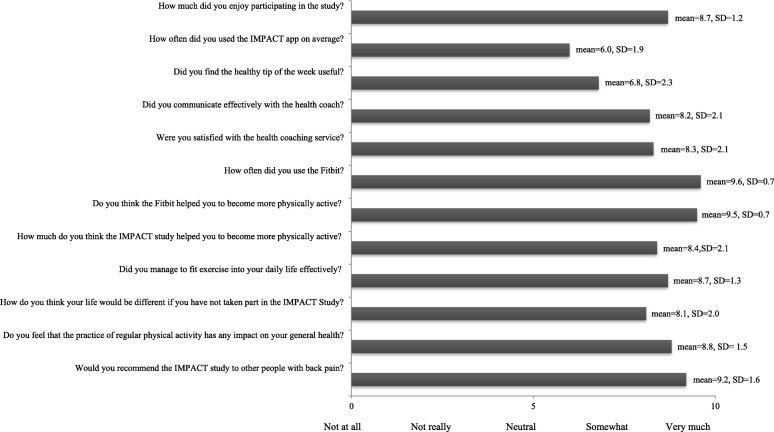
Experience of the intervention participants from the IMPACT Study. Each question reported in the figure required a response on 0 to 10 Likert scale where higher scores indicate higher satisfaction. In the figure, each bar corresponds to the mean score for each question displayed in the left-hand side of the figure

We aimed to interview a minimum of 20 participants. Of the 31 participants from the intervention group who completed the 6-month follow-up, 24 were interviewed about their experiences with the intervention. The other seven participants completed the follow-up before ethics was granted for the interviews and therefore were not invited to participate. Overall, participants were satisfied with the intervention (mean = 8.7 on the 0 to 10 satisfaction scale, where higher scores indicate higher satisfaction) (Fig. 3). Participants were presented with the main features of the study (e.g. the *Fitbit*, the health coaching, the IMPACT app or receiving the weekly surveys) and were asked to rank the level of preference. The aspect of the intervention that participants enjoyed most was using the *Fitbit* (53%), followed by the health coaching (29%). The aspect they least enjoyed was receiving the weekly surveys (6%). Most intervention group participants (*n* = 23, 96%) reported wearing the *Fitbit* every day or most days during the intervention and felt that it was useful to motivate them to be more active, with most participants (71%) engaging in physical activity for four or more times per week. Furthermore, most participants (88%) believed the amount of contact with the health coach (mean = 11 sessions, SD ± 2) was appropriate and reported that coaching sessions were helpful for encouraging them to be physically active. No adverse events were reported.

### Intervention impact on clinical outcomes

#### Primary outcomes

**Fig. 4:**
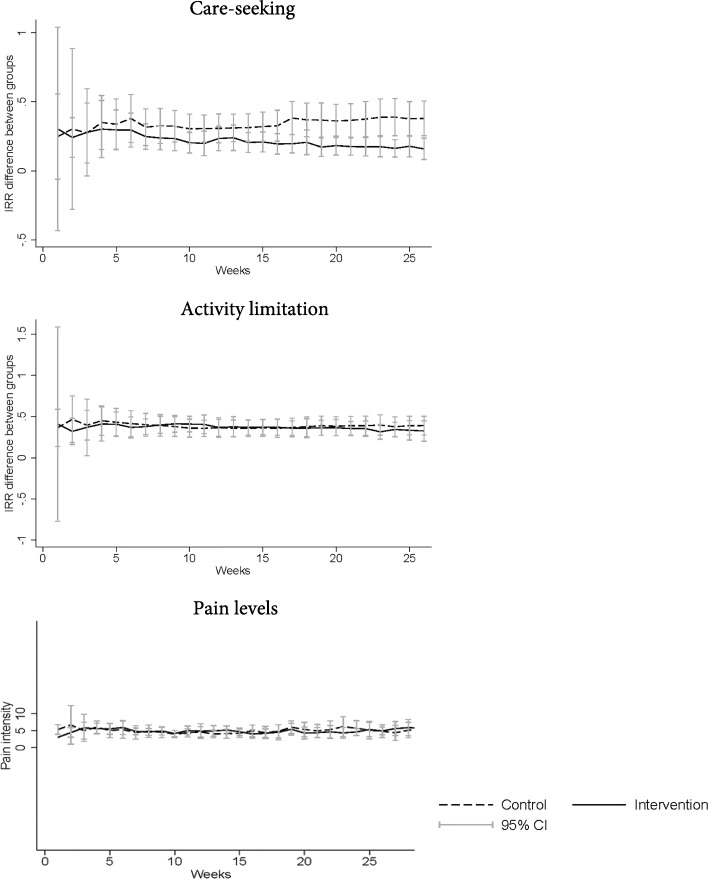
Weekly difference between groups in care-seeking, activity limitation and low back pain levels throughout the study

Data on the primary outcomes are presented in Table 3. Primary outcomes were collected weekly for 6 months in total. Overall, the average number of care-seeking episodes/person per group was higher in the control group when compared to the intervention group (mean = 6.3, SD ± 7.8, mean = 3.1, SD ± 4.6, respectively). However, this difference between groups did not reach statistical significance. The Poisson regression analysis showed that participants in the intervention group, on average, and across the follow-up period, had a 38% non-significant reduced rate of care-seeking (IRR: 0.62, 95% CI: 0.32 to 1.18, *p* = 0.14) compared to control group. Regarding the group effect over time, there was a weekly reduction rate of care-seeking of 3% in the intervention group (IRR: 0.97, 95% CI: 0.93 to 1.01, p = 0.14) (Fig. 4). There were no between groups differences for activity limitation during the follow-up period. Regarding the group effect over time, there was a non-significant weekly reduction of 1% in the rate of activity limitation in the intervention group (IRR: 0.99, 95% CI: 0.96 to 1.02, *p* = 0.66). For pain levels there were no between groups differences across the follow-up period and we did not find any group effect over time.

**Table 3:** Effects of intervention for primary outcomes (with 95% confidence intervals)

Primary outcomes	Group Effect					Group Effect Over Time*		
	n	Obs	IRR/Coef.^β^	95% CI	p	IRR/Coef. ^β^	95% CI	p
Care-seeking^a^	57	616	0.62	0.32–1.18	0.147	0.97	0.93–1.01	0.144
Activity limitation^b^	57	622	1.04	0.59–1.83	0.868	0.99	0.96–1.02	0.660
Pain levels^c^	57	605	0.24	−0.76–1.25	0.635	−0.01	−0.04–0.01	0.303

^*^Multilevel mixed-effects and interaction with time variable for the outcomes collected weekly

^β^Incidence-Rate Ratio (IRR) refer to care-seeking and activity limitation. Pain is presented as coefficient (Coef)

^a^Care-seeking due to LBP in the last week (yes or no)

^b^Activity limitation due to LBP in the last week (yes or no)

^c^Low Back Pain level in the last week measured with the numerical rating scale (0-10); Coef.: coefficient; IRR: Incidence Risk Ratio; CI: Confidence Interval

#### Secondary outcomes

Table 4 shows the group data at baseline and 6 months for the secondary outcomes. Participants in the intervention group self-reported more walking at follow-up (assessed with the IPAQ) compared with the control group (183.1 min per week; 95% CI: 48.5 to 317.7, *p* = 0.009) and a higher proportion of the intervention group attained their physical activity goals at 6 months compared to the control group (OR: 6.5; 95% CI: 1.9 to 22.5, *p* = 0.003). There were no between-group differences for self-reported MVPA, or objectively assessed physical activity assessed.

**Table 4:** Mean (SD) of outcomes by group at baseline and follow-ups and effects of intervention

Outcomes	Intervention Baseline	Intervention Follow-up	Control Baseline	Control Follow-up	Intervention vs Control*	Intervention Baseline	
	n = 34	*n* = 31	n = 34	*n* = 24	Coef./OR^β^	95% CI	p
Pain intensity, score/10^a^	5.3 (1.9)	3.8 (2.4)	5.1 (1.4)	4.0 (3.4)	−0.14	−1.34–1.06	0.815
Disability, score/24^b^	8.9 (5.4)	5.7 (5.3)	9.0 (6.1)	6.0 (5.7)	−0.47	−3.13–2.18	0.722
Self-reported walking, min/week^c^	340.3 (688.9)	453.0 (942.5)	250.8 (221.2)	254.5 (390.8)	183.1	48.53–317.68	0.009
Self-reported moderate PA, min/week^c^	109.7 (379.1)	60.9 (96.1)	93.5 (273.0)	159.7 (343.5)	61.0	−46.05–168.12	0.256
Self-reported vigorous PA, min/week^c^	89.4 (363.5)	77.3 (174.1)	35.3 (165.8)	71.2 (163.3)	50.5	−63.83–164.81	0.377
Objective light PA, min/week^d^	1984.9 (712.2)	2065.7 (529.5)	1936.7 (655.5)	1941.2 (546.2)	133.5	− 169.6–436.6	0.378
Objective MVPA, min/week^e^	202.2 (152.4)	187.7 (138.5)	200.5 (166.2)	169.2 (131.8)	35.7	−38.2–109.6	0.334
Step count/ week^f^	51,613 (27007)	51,659 (25389)	50,684 (29072)	49,141 (24883)	6301	0.347–19,719	0.347
Goal attainment^g^, number (%)^β^	–	20 (65)	–	5 (22)	6.54	1.90–22.48	0.003

^*^Between-group differences at 6 months, adjusted for baseline values, with 95% confidence intervals (CI)

^β^Between group differences for goal attainment is presented as Odds Ratio (OR), all the other outcomes are presented as coefficients (Coef)

^a^Pain intensity measured with the numerical rating scale (0–10)

^b^Disability measured with the Rolland-Morris Disability Questionnaire (0–24)

^c^Total minutes of physical activity per week measured with the International Physical Activity Questionnaire (IPAQ)

^d^Total minutes of light physical activity objectively measured with the Actigraph

^e^Total minutes of moderate-to-vigorous physical activity (MVPA) objectively measured with the Actigraph

^f^Total steps taken per week objectively measured with the Actigraph

^g^Goal attainment measured with the Goal Attainment Scale (GAS) PA = physical activity. Coef coefficient; OR: Odds Ratio

## Discussion

### Feasibility

Results from this study indicate that a physical activity intervention for people with chronic LBP that involves health coaching, activity trackers, and m-Health over 6 months is feasible and acceptable by the target population. Our results also indicate some impact of the intervention on the primary outcome of care-seeking and the secondary outcomes of self-reported walking and physical activity goal attainment.

Ease of participant recruitment differed in the hospital setting compared with the general community, with more participants recruited from the general community in a shorter period compared with the public hospitals over a longer period. This discrepancy is likely due to the culturally diverse background of patients screened at the hospitals when compared to the general community, with sufficient English language skills being required for study enrolment. Australia is known to have a large culturally diverse migrant population, and this can present challenges for clinical trial recruitment and intervention delivery. [64] To overcome this limitation, translation services could be utilised to facilitate recruitment and study implementation in culturally diverse hospital sites.

Further potential challenges identified in this study should be considered for the implementation of a full-scale trial. One aspect that deserves attention is the high number of drop-outs in the control group, which at 19% was lower than the anticipated 35% previously reported in the trial protocol. [38] A lower drop-out rate in the control group may be achieved with the inclusion of a sham advice group, with the same amount of therapist interaction (phone calls) as the intervention group. Also, the weekly data collection resulted in a large amount of missing data, which could have underpowered our study to detect intervention effects on care-seeking and pain intensity. To reduce the amount of missing data, primary outcomes could be collected less frequently (for example on a fortnightly basis) to minimise study burden on participants. Further, this study was associated with a 42% rate of loss to follow-up in the physical activity outcome, which in part reflects the requirement to wear the accelerometer for seven days. [65] To increase compliance with the *Actigraph* protocol, participants could be contacted during the 7-day period of data collection and be reminded to use the *Actigraph* for at least 10 h a day during that week. Lastly, in order to more accurately measure the impact of the intervention on physical activity levels, in a future full-scale trial the inclusion criteria should take into consideration participants’ level of physical activity at baseline. As the intervention targeted increasing participants’ physical activity levels, people that are already exceeding physical activity guidelines at baseline should be excluded and recruitment should focus in targeting those recognised as inactive.

### Clinical impact

Although this pilot clinical trial was not powered to detect a difference in healthcare utilisation, the direction and magnitude of findings suggest a possible beneficial effect of the intervention to reduce care-seeking over time. Also, we observed a significant increase in the amount of self-reported walking in the intervention group compared to the control group, but not in MVPA assessed either by self-report or objective methods. Furthermore, a higher number of participants in the intervention group (65%) achieved their physical activity goals as compared to the control group (22%), indicating a beneficial impact on behaviour.

To the best of our knowledge, this is the first study that has investigated the effect of a health coaching physical activity intervention not only in decreasing pain and activity limitation but also in decreasing care-seeking in people with chronic LBP after discharge from physiotherapy treatment. To date, there is insufficient evidence of the effect of health coaching-based interventions for decreasing pain and activity limitations in people with LBP. [66, 67] Most published trials have not clearly defined the principles on which the health coaching is based (e.g. behaviour change theory, motivational interviewing), or the methods by which coaches are trained (e.g. certified courses, amount of training) to ensure the treatment is delivered as intended. [68] This variability in study settings challenges the comparison of our findings with previous studies. Our study clearly defined the intervention, which involved health coaching, based on goal setting, motivational interviewing and behaviour change theory, which has been identified by a recent systematic review [69] to be the most effective approach to improve health outcomes.

Recent research has suggested that chronic LBP interventions should prioritise self-management to reduce healthcare utilisation rather than pain intensity, given that pain levels are not significantly sensitive to change over time. [70] However, the main challenge to effective self-management of LBP is limited adherence to physical activity and lifestyles that are most likely to reduce the physical and emotional “triggers” that aggravate symptoms of LBP. [71] Although we did not observe between-group differences in pain or activity limitation in our study, we detected a trend of reduction in care-seeking and a significant increase in walking and physical activity goal attainment in favour of the intervention group, which are clinically valuable. To detect a clinically meaningful between-group difference, a full-scale randomised controlled trial could estimate the sample size based on care-seeking rates observed in this trial.

With regard to physical activity, there was a significant increase in walking time in the intervention group compared to the control group. Most recent guidelines support walking as an essential component of management for chronic LBP. [72] In our study, intervention participants were encouraged to use the *Fitbit*, which has been found to be effective in increasing walking in people with LBP. [37] The *Fitbit* was used as a feedback tool and participants were encouraged to walk and set goals related to step count in addition to the other preferred activities. Additionally, we found a significant between-group difference in goal attainment at 6 months in favour of the intervention group. This may be explained by the fact that many participants set goals related to walking, which increased significantly in the intervention group. Moreover, many participants set goals associated with specific structured activities, such as yoga, Pilates or swimming, which are activities that are less sensitive to be registered with the *Actigraph*. [44, 73] Consequently, it is likely that this device was not sufficiently sensitive to detect increases in participation for these types of activities.

### Strengths and limitations

A key strength of this trial is its low risk of bias due to central randomisation and allocation concealment, blinded outcome assessment, intention-to-treat analysis, and pre-publication of a study protocol and statistical analysis plan. [38] We conducted this study in accordance with CONSORT guidelines and followed the prospectively registered protocol. Further study strengths include the availability of weekly collected data on LBP intensity and care-seeking gathered during the time of the intervention. Additionally, we used objective physical activity measures, which are more accurate compared to self-reported measurements. [74]

A potential limitation is that our intervention included several pragmatically delivered components, such as health coaching, which included a range of techniques (i.e. goal setting, motivational interviewing), activity trackers, and mobile technology (IMPACT App). As yet, it is unclear which techniques or components of the intervention are effective or not. However, this is a pilot trial, and therefore the main aim was to evaluate the feasibility and preliminary efficacy of outcomes and its impact on healthcare utilisation. Moreover, we did not evaluate frequently reported cognitive and emotional factors such as fear of movement, catastrophising, and anxiety, which impact on pain and activity limitation. [75] A closer exploration of these cognitive behavioural factors and their impact on pain and disability is needed. Another potential limitation is that there was no follow-up contact with the control group, apart from the weekly survey sent via mobile text message or e-mail. It can be argued that since the intervention group received greater contact from the research team, this contextual factor could have influenced the results. We aim to include a sham advice group in a future full-scale trial to minimise this issue.

## Conclusion

This pilot trial provides proof of concept, preliminary evidence of the success of the intervention, and evidence that this patient-centred physical activity intervention (supported by health coaching and m-Health technology) is feasible in a population with LBP. The intervention was associated with increased mobility goal attainment and walking volume at 6 months and may reduce rates of additional care-seeking after treatment discharge. This result, however, should be interpreted cautiously due to underpowered analysis. If these effects are evident in a full-scale trial, this novel model of care may be an effective management strategy for patients with chronic LBP after treatment discharge, and the public health implications would be substantial.

## Additional file

Additional file 1: Low back pain weekly survey used to collect primary outcomes. (DOCX 20 kb)

## Abbreviations

BMI: Body Mass Index
CI: Confidence Interval
DASS: Depression Anxiety Stress Scale
FABQ: Fear Avoidance Belief Questionnaire
GAS: Goal Attainment Scale
GP: General Practitioner
IPAQ: International Physical Activity Questionnaire
IQR: Inter Quartile Range
IRR: Incidence Rate Ratio
LBP: Low Back Pain
MVPA: Moderate-to-Vigorous Physical Activity
NRS: Numerical Rating Scale
OR: Odds Ratio
PSQI: Pittsburgh Sleep Quality Index
RMDQ: Roland–Morris Disability Questionnaire
SD: Standard Deviation
TIDieR: Template for Intervention Description and Replication
WHO: World Health Organization

## References

[1] Hartvigsen J, et al. What low back pain is and why we need to pay attention. Lancet. 2018. 10.1016/S0140-6736(18)30480-X 29573870

[2] Disease GBD, Injury I, Prevalence C (2016). Global, regional, and national incidence, prevalence, and years lived with disability for 310 diseases and injuries, 1990-2015: a systematic analysis for the global burden of Disease study 2015. Lancet.

[3] Marras WS (2007). Low back pain recurrence in occupational environments. Spine (Phila Pa 1976).

[4] Skargren, E.I., P.G. Carlsson, and B.E. Oberg, One-year follow-up comparison of the cost and effectiveness of chiropractic and physiotherapy as primary management for back pain. Subgroup analysis, recurrence, and additional health care utilization*.* Spine (Phila Pa 1976), 1998. 23(17): p. 1875–1883; discussion 1884. 10.1097/00007632-199809010-00016 9762745

[5] Stanton TR (2008). After an episode of acute low back pain, recurrence is unpredictable and not as common as previously thought. Spine (Phila Pa 1976).

[6] Steenstra IA (2005). Prognostic factors for duration of sick leave in patients sick listed with acute low back pain: a systematic review of the literature. Occup Environ Med.

[7] Kent PM, Keating JL (2005). The epidemiology of low back pain in primary care. Chiropr Osteopat.

[8] Thelin A, Holmberg S, Thelin N (2008). Functioning in neck and low back pain from a 12-year perspective: a prospective population-based study. J Rehabil Med.

[9] Ferreira ML (2007). Comparison of general exercise, motor control exercise and spinal manipulative therapy for chronic low back pain: a randomized trial. Pain.

[10] Chou R (2017). Nonpharmacologic therapies for low Back pain: a systematic review for an American College of Physicians Clinical Practice Guideline. Ann Intern Med.

[11] Airaksinen O (2006). Chapter 4. European guidelines for the management of chronic nonspecific low back pain. Eur Spine J.

[12] Kongsted A (2015). Patients with low back pain had distinct clinical course patterns that were typically neither complete recovery nor constant pain. A latent class analysis of longitudinal data. Spine J.

[13] Enthoven P, Skargren E, Oberg B (2004). Clinical course in patients seeking primary care for back or neck pain: a prospective 5-year follow-up of outcome and health care consumption with subgroup analysis. Spine (Phila Pa 1976).

[14] Jack K (2010). Barriers to treatment adherence in physiotherapy outpatient clinics: a systematic review. Man Ther.

[15] Shiri R, Falah-Hassani K (2017). Does leisure time physical activity protect against low back pain? Systematic review and meta-analysis of 36 prospective cohort studies. Br J Sports Med.

[16] Pinto RZ (2014). Self-reported moderate-to-vigorous leisure time physical activity predicts less pain and disability over 12 months in chronic and persistent low back pain. Eur J Pain.

[17] Leeuw M (2007). The fear-avoidance model of musculoskeletal pain: current state of scientific evidence. J Behav Med.

[18] Lin CW (2011). Relationship between physical activity and disability in low back pain: a systematic review and meta-analysis. Pain.

[19] Liddle SD, Baxter GD, Gracey JH (2007). Chronic low back pain: patients' experiences, opinions and expectations for clinical management. Disabil Rehabil.

[20] May S (2007). Patients' attitudes and beliefs about back pain and its management after physiotherapy for low back pain. Physiother Res Int.

[21] Macedo LG, Bostick GP, Maher CG (2013). Exercise for prevention of recurrences of nonspecific low back pain. Phys Ther.

[22] O'Sullivan P (2012). It's time for change with the management of non-specific chronic low back pain. Br J Sports Med.

[23] Vibe Fersum K (2013). Efficacy of classification-based cognitive functional therapy in patients with non-specific chronic low back pain: a randomized controlled trial. Eur J Pain.

[24] Hill JC (2011). Comparison of stratified primary care management for low Back pain with current best practice (STarT Back): a randomised controlled trial. Lancet.

[25] Kunstler BE (2018). Physiotherapist-led physical activity interventions are efficacious at increasing physical activity levels: a systematic review and meta-analysis. Clin J Sport Med.

[26] Oliveira JS (2017). What is the effect of health coaching on physical activity participation in people aged 60 years and over? A systematic review of randomised controlled trials. Br J Sports Med.

[27] Huffman MH (2009). HEALTH COACHING: a fresh, new approach to improve quality outcomes and compliance for patients with chronic conditions. Home Healthc Nurse.

[28] Kivela K (2014). The effects of health coaching on adult patients with chronic diseases: a systematic review. Patient Educ Couns.

[29] McDonough SM (2013). Pedometer-driven walking for chronic low back pain: a feasibility randomized controlled trial. Clin J Pain.

[30] Eakin EG (2007). Telephone interventions for physical activity and dietary behavior change: a systematic review. Am J Prev Med.

[31] Lichtenstein E (1996). Telephone counseling for smoking cessation: rationales and meta-analytic review of evidence. Health Educ Res.

[32] Allen KD (2010). Telephone-based self-management of osteoarthritis: a randomized trial. Ann Intern Med.

[33] Krishna S, Boren SA, Balas EA (2009). Healthcare via cell phones: a systematic review. Telemed J E Health.

[34] Cole-Lewis H, Kershaw T (2010). Text messaging as a tool for behavior change in disease prevention and management. Epidemiol Rev.

[35] Bravata DM (2007). Using pedometers to increase physical activity and improve health: a systematic review. JAMA.

[36] Moessner M, Schiltenwolf M, Neubauer E (2012). Internet-based aftercare for patients with back pain-a pilot study. Telemed J E Health.

[37] Mansi S (2014). A systematic review of studies using pedometers as an intervention for musculoskeletal diseases. BMC Musculoskelet Disord.

[38] Amorim AB (2016). Integrating Mobile health and physical activity to reduce the burden of chronic low back pain trial (IMPACT): a pilot trial protocol. BMC Musculoskelet Disord.

[39] Schulz KF (2011). CONSORT 2010 statement: updated guidelines for reporting parallel group randomised trials. Int J Surg.

[40] Yamato TP (2016). How completely are physiotherapy interventions described in reports of randomised trials?. Physiotherapy.

[41] Gorman E (2014). Accelerometry analysis of physical activity and sedentary behavior in older adults: a systematic review and data analysis. Eur Rev Aging Phys Act.

[42] Hashimoto Y (2018). Association between objectively measured physical activity and body mass index with low back pain: a large-scale cross-sectional study of Japanese men. BMC Public Health.

[43] Plasqui G, Westerterp KR (2007). Physical activity assessment with accelerometers: an evaluation against doubly labeled water. Obesity (Silver Spring).

[44] Schrack JA (2016). Assessing daily physical activity in older adults: unraveling the complexity of monitors, measures, and methods. J Gerontol A Biol Sci Med Sci.

[45] Wood AC (2008). Actigraph data are reliable, with functional reliability increasing with aggregation. Behav Res Methods.

[46] Conway J, Tomkins CC, Haig AJ (2011). Walking assessment in people with lumbar spinal stenosis: capacity, performance, and self-report measures. Spine J.

[47] Brown WJ, Bauman A, Bull FC, Burton NW. Development of Evidence-based Physical Activity Recommendations for Adults (18-64 years). Report prepared for the Australian Government Department of Health. 2012.

[48] Schaller A, Froboese I (2014). Movement coaching: study protocol of a randomized controlled trial evaluating effects on physical activity and participation in low back pain patients. BMC Musculoskelet Disord.

[49] Matthews CE (2002). Sources of variance in daily physical activity levels as measured by an accelerometer. Med Sci Sports Exerc.

[50] Choi L (2011). Validation of accelerometer wear and nonwear time classification algorithm. Med Sci Sports Exerc.

[51] de Vet HC (2002). Episodes of low back pain: a proposal for uniform definitions to be used in research. Spine (Phila Pa 1976).

[52] Von Korff M, Jensen MP, Karoly P (2000). Assessing global pain severity by self-report in clinical and health services research. Spine (Phila Pa 1976).

[53] Stratford PW, Riddle DL (2016). A Roland Morris disability questionnaire target value to distinguish between functional and dysfunctional states in people with low Back pain. Physiother Can.

[54] Craig CL (2003). International physical activity questionnaire: 12-country reliability and validity. Med Sci Sports Exerc.

[55] Jaeschke L (2017). 24 h-accelerometry in epidemiological studies: automated detection of non-wear time in comparison to diary information. Sci Rep.

[56] Choi L (2012). Assessment of wear/nonwear time classification algorithms for triaxial accelerometer. Med Sci Sports Exerc.

[57] Jaeschke L (2018). Variability and reliability study of overall physical activity and activity intensity levels using 24 h-accelerometry-assessed data. BMC Public Health.

[58] Colley RC (2011). Physical activity of Canadian adults: accelerometer results from the 2007 to 2009 Canadian health measures survey. Health Rep.

[59] Organization (2010). W.H., Global recommendations on physical activity for health*.*, W.H. Organization.

[60] Turner-Stokes L (2009). Goal attainment scaling (GAS) in rehabilitation: a practical guide. Clin Rehabil.

[61] Waddell G (1993). A fear-avoidance beliefs questionnaire (FABQ) and the role of fear-avoidance beliefs in chronic low back pain and disability. Pain.

[62] Lovibond SH, Lovibond PF (1995). Manual for the depression and anxiety scales.

[63] Buysse DJ (1989). The Pittsburgh sleep quality index: a new instrument for psychiatric practice and research. Psychiatry Res.

[64] Foster NE, et al. Prevention and treatment of low back pain: evidence, challenges*,* and promising directions. Lancet. 2018. 10.1016/S0140-6736(18)30489-6 29573872

[65] Kocherginsky M (2017). Correction: measuring physical activity with hip Accelerometry among U.S. older adults: how many days are enough?. PLoS One.

[66] Holden J, Davidson M, O'Halloran PD (2014). Health coaching for low back pain: a systematic review of the literature. Int J Clin Pract.

[67] Mansell G, Hall A, Toomey E (2016). Behaviour change and self-management interventions in persistent low back pain. Best Pract Res Clin Rheumatol.

[68] Keogh A (2015). A review of behaviour change theories and techniques used in group based self-management programmes for chronic low back pain and arthritis. Man Ther.

[69] Wolever RQ (2013). A systematic review of the literature on health and wellness coaching: defining a key behavioral intervention in healthcare. Glob Adv Health Med.

[70] Beattie PF, Silfies SP (2015). Improving long-term outcomes for chronic low back pain: time for a new paradigm?. J Orthop Sports Phys Ther.

[71] Steffens D (2014). Clinicians' views on factors that trigger a sudden onset of low back pain. Eur Spine J.

[72] Qaseem A (2017). Noninvasive treatments for acute, subacute, and chronic low Back pain: a clinical practice guideline from the American College of Physicians. Ann Intern Med.

[73] Tucker JM, Welk GJ, Beyler NK (2011). Physical activity in U.S.: adults compliance with the physical activity guidelines for Americans. Am J Prev Med.

[74] Celis-Morales CA (2012). Objective vs. self-reported physical activity and sedentary time: effects of measurement method on relationships with risk biomarkers. PLoS One.

[75] Woby SR (2004). Are changes in fear-avoidance beliefs, catastrophizing, and appraisals of control, predictive of changes in chronic low back pain and disability?. Eur J Pain.

