# Supplementary material for: Integrating Mobile-health, health coaching, and physical activity to reduce the burden of chronic low back pain trial (IMPACT): a pilot randomised controlled trial
Source: BMC Musculoskelet Disord. 2019 Feb 11;20:71. doi: 10.1186/s12891-019-2454-y (PMC6371593; doi:10.1186/s12891-019-2454-y)
Supplement: Supplementary file 1 — Low back pain weekly survey used to collect primary outcomes. (DOCX 20 kb) [file 12891_2019_2454_MOESM1_ESM.docx]

**Additional file 1: Low back pain weekly survey**

1. Have you had low back pain in the last 7 days?

( ) Yes

( ) No

*Observation: the following questions will only appear if the participant has answered “Yes” for the previous question.*

1. Please indicate on average what was the intensity of your low back pain on a scale from 0 to 10, where 0 means “no pain” and 10 means “the worst pain imaginable”, over the last 7 days.*

| 0 | 1 | 2 | 3 | 4 | 5 | 6 | 7 | 8 | 9 | 10 |
| --- | --- | --- | --- | --- | --- | --- | --- | --- | --- | --- |
| No pain |  |  |  |  | Moderate Pain |  |  |  |  | Worst possible pain |

1. Was the low back pain bad enough to limit your usual activities in the **last 7 days**?*

( ) Yes (the following options will only appear if they answer “yes”)

( ) No (go to next question)

( ) Work

( ) Socializing

( ) Sports

( ) Hobbies

( ) Intimacy

( ) Chores

1. Have you sought any treatment for this low back pain in the last 7 days?*

( ) Yes (the following options will only appear if they answer “yes”)

( ) No (go to next question)

( ) GP

( ) Physiotherapist

( ) Chiropractor

( ) Emergency department

( ) Surgical procedure

( ) Other Please specify:

Trials used written infor-

mation, discussion sessions, and audiovisual resources

(i.e., audiotape, videotape, or web site) as self-management

strategies.

*Weekly pain levels outcome was based on question 2; weekly disability outcome was based on question 3; weekly care-seeking outcome was based on question 4.
